# Supplementary material for: A qualitative study about how families coped with managing their well-being, children’s physical activity and education during the COVID-19 school closures in England
Source: PLoS One. 2022 Dec 22;17(12):e0279355. doi: 10.1371/journal.pone.0279355 (PMC9778504; doi:10.1371/journal.pone.0279355)
Supplement: S1 Text — (DOCX) [file pone.0279355.s001.docx]

**Text S1: Interview guide**

**How are parents and children coping with the school closures?**

**Broad Objectives (15 mins)**

1. ***To gain insight into the home environment of the children***

- (TURN ON RECORDER) Hello, is this xxx? My name is *xxx*, thank you for agreeing to take part in this interview where we will be discussing how parents and children are coping with the school closures because of the coronavirus pandemic.
- This interview is being recorded this is to help keep the accuracy of what is discussed but I will remind you that what you say will be anonymised in our reporting and kept confidential
- You also have the right to withdraw from this interview.
- The purpose of this interview is for me to understand your experiences and therefore please don’t be concerned with the right or wrong answer but for you to provide details on your individual experience. Please also share any information that you feel may be relevant to the topic, but I may not have asked.
- Are there any questions that you have about the information sheet? Or before we start?
- *OK, thank you before we start can I clarify an item on the screener, you mentioned xxx although xxx*

So, you have a xxx year old and xxx, is that correct? Would you mind telling me a bit about your children? And what do they like doing? (IF PAUSE) What are their hobbies? OR what was the last film you all watched together?

Would you be able describe your children’s activities on a usual weekday and weekend before the schools closed?

And what does a weekday and weekend look like now?

Does it seem different to how they would spend the school summer holidays?

*You mentioned in the screener that you had the option for your children to continue to attend school as you are a key worker. Are you able to explain what was important to you in deciding to keep your children at home?*

**Guidance (50 mins)**

1. ***To understand how the government advice is being followed***
2. ***To learn children’s interactions outside the home***
3. ***Identify the challenges, benefits and coping in the current situation***

Thank you for letting me know a bit about your family. We are now going to move onto the next section of the interview about managing during the school closures.

Have you been able to stay at home? How have you found it? *Only go outside for food, health reasons or work (but only if you cannot work from home)*

- Where have you gone? How often?
- Were your children with you?

Would you say your children have been able to social distance when outside? *2 meters (6ft) away from other people*

How have your children’s social interactions changed?

Have you been able to keep your children from interacting with other people?

- How have you managed this? Have there been any challenges?
- **(IF NO)** Can you tell me who they have been interacting with and what they have been doing?
- How often and how long?

Are you aware of what a vulnerable person may be? *Aged 70 or older, or under 70 with an underlying health condition*

Have your children been in contact with any vulnerable people?

How have your children been preventing the spread of coronavirus inside and outside your home?

How do you feel about your children being educated at home?

How much education are your children receiving?

Are you able to monitor their work?

Have your children been exercising?

Can you tell me what your children have found to be the most challenging about the school closures?

- How have your children been adapting to those challenges?

Have you noticed any changes in your children’s mood or behaviour?

Can you think of any aspect of your children’s life that may have benefited from the current situation?

Have your children been able to stick to the guidance on hand hygiene? *20 sec, soap, get home, cover mouth and nose with tissue or sleeve when cough or sneeze, used tissues in the bin and wash hands afterwards*

How much do you feel your children understand about coronavirus?

Has your child /ren had coronavirus or coronavirus symptoms? Either a *High temperature or new, continuous cough*
**(IF NO)** Would you find it difficult to cope if they did have to self-isolate? Or you? Would you be able to get the essentials that you need?

Can you describe what happened?

During that time, what were your main concerns?

Does your child / ren have any existing mental or physical disabilities?
**🡪** Do you think their needs are being met?

Have you considered taking your children to get medical attention…(*GP or hospital*)? What happened? *Did you take them? How would you feel if there was an accident and you had to take them?*

Thinking about the social distancing changes or hygiene behaviours that we have discussed, what were the main reasons for making those changes?

Have you changed the media content you view surrounding coronavirus? When and which?

Other than what we have already discussed, is there a preventative measure that you would like make but you feel you are unable to?

**Attitudes (25mins)**

1. ***To understand the attitudes to the lock-down and school closures***
2. ***To recognise future changes in behaviour***

Thank you for your responses to those questions. This is the last section of the interview which is a few questions on your views of the school closures.

Can you think back to when you found out that schools were being closed, what were your initial thoughts on hearing the announcement?

Do you still think those things?

What do you feel about the lockdown measures that are currently in place?

How long do you think the lockdown will last?
 **🡪** do you feel you will be able to last that long?

Has this experience changed the way you think about your children being ill?

Are there any changes in behaviour that your children have started to do since the lockdown, that you will try to continue to do once it has finished?

What are your thoughts on a partial school re-opening?... *For example, specific children are allowed in school for specific days of the week or times of day.*

Do you have any worries about sending your children back to school?

Do you feel your children are experiencing a lot of difficultly with the schools being closed
 **🡪** Are you able to suggest why that might be?

**Debrief**

That brings us to the end of the interview. Is there anything that you would like to add or clarify?

How did you find the interview?

Well thank you for your time, I have learnt a lot of valuable information especially in regard to xxx Angelfish will be in contact to organise your thank you incentive.

- Enjoy the rest of your day

**TURN OFF RECORDER**
